# Mosquitoborne Infections after Hurricane Jeanne, Haiti, 2004

**DOI:** 10.3201/eid1302.061132

**Published:** 2007-02

**Authors:** Mark E. Beatty, Elizabeth Hunsperger, Earl Long, Julia Schürch, Seema Jain, Rom Colindres, Gerald Lerebours, Yves-Marie Bernard, James Goodman Dobbins, Mathew Brown, Gary G. Clark

**Affiliations:** * Centers for Disease Control and Prevention, San Juan, Puerto Rico, USA; † Centers for Disease Control and Prevention, Atlanta, Georgia, USA; ‡ Médecins Sans Frontières, Belgium; § John Snow Incorporated, Port-Au-Prince, Haiti; ¶ Centers for Disease Control and Prevention, Port-Au-Prince, Haiti; # Pan American Health Organization, Port-Au-Prince, Haiti; ** Agricultural Research Service, Gainesville, Florida, USA

**Keywords:** Mosquitoborne encephalitis, West Nile fever, dengue, malaria, natural disasters, Haiti, dispatch

## Abstract

After Hurricane Jeanne in September 2004, surveillance for mosquitoborne diseases in Gonaïves, Haiti, identified 3 patients with malaria, 2 with acute dengue infections, and 2 with acute West Nile virus infections among 116 febrile patients. These are the first reported human West Nile virus infections on the island of Hispaniola.

Hurricane Jeanne caused large-scale devastation in Gonaïves, Haiti, on September 18, 2004. The US Department of Health and Human Services assisted the Haitian Ministry of Health by conducting a rapid field assessment of health-related issues. Among the actions recommended by the team was immediate epidemiologic assistance from the Centers for Disease Control and Prevention (CDC) to reinforce and expand epidemiologic surveillance to identify as early as possible any emerging epidemic or community health problems. Concern was raised that the combination of flooding, loss of shelter, and destruction of infrastructure would result in an outbreak of mosquitoborne diseases. We conducted surveillance to assess the extent of mosquitoborne diseases and monitor for outbreaks of these diseases.

## The Study

We established laboratory-based fever surveillance at the 3 clinics providing healthcare in Gonaïves after the passage of Hurricane Jeanne. Febrile patients (core temperature ≥38.5°C when first assessed) were asked to provide blood for a serum sample and thick and thin malaria smears. The attending physician recorded each patient’s medical history, conducted a physical examination, and reported the discharge diagnosis and the therapy that was provided. We asked patients to return in 2 weeks so that a convalescent-phase serum sample could be collected.

Malaria smears were stained and read by using standard methods (*1*) at CDC. To diagnose dengue, we used nested PCR and the TaqMan assay to detect dengue viral RNA in serum samples obtained ≤5 days after onset of symptoms (*2*,*3*). In addition, we used an immunoglobulin M (IgM) antibody-capture (MAC)–ELISA to detect anti-dengue IgM antibodies in all serum specimens (*4*) at CDC. A result was considered positive when optical density, after comparison to negative serum and control antigen, was >0.20. All serum specimens were also tested for the presence of IgG antibodies to determine previous exposure to flaviviruses by using an IgG ELISA. In paired samples, a full titration of 4-fold dilutions of serum was used. The endpoint titration of IgG was determined to assess seroconversion (*5*). Each plate was compared with a negative control serum specimen. Because of cross-reactivity between anti-flavivirus antibodies, we used a microsphere-based immunoassay (MIA) with a quadratic discrimination analysis (*6*) and a plaque reduction neutralization test (PRNT) to distinguish between infecting flaviviruses. For the PRNT, serial dilutions of heat-inactivated serum were incubated with defined amounts of West Nile, Saint Louis encephalitis, and dengue viruses 1–4 for 2 hours at room temperature. The nonneutralized viral fraction was subsequently adsorbed onto a monolayer of Vero cells for 1 hour. The resultant plaques were counted and compared with results for the control virus with no serum. The endpoint of the titration was the highest dilution of serum that reduced the number of plaques 90% compared with the control results.

From November 15 through December 22, 2004, 116 acutely febrile patients were identified and included in our surveillance. Ages ranged from 4 months to 71 years (median 4 years); 52% were female. All patients lived in Gonaïves. Seventy-one patients (61%) appeared for treatment with a chief complaint of fever with cough, 35 (30%) had fever with no apparent source, 6 (5%) reported fever with diarrhea, and 4 (3%) reported fever with rash. Patients sought treatment a median of 3 days after the onset of fever (range 0–28 days). In addition to fever, the most commonly reported symptoms were cough (77, 66%), abdominal pain (57, 49%), and headache (56, 48%). Thirty-nine patients (34%) had at least 1 clinical sign of dehydration; 16 patients (14%) were hypotensive on physical examination. No patients were jaundiced or had spontaneous hemorrhage. The most common clinical diagnoses were upper respiratory infection (35, 30%), malaria (34, 29%), pneumonia (21, 18%), and typhoid fever (13, 11%). No cases of dengue fever were suspected. Fifty-eight patients (50%) were treated with oral antimicrobial drugs; 13 patients (11%) were prescribed chloroquine, and 2 patients (2%) received an antihelminthic drug. All cases of suspected malaria were diagnosed by patients’ clinical symptoms. Suspected tuberculosis was confirmed in 1 patient by a positive sputum smear. None of 116 patients was admitted to a hospital.

Of the 116 thick and thin smears, 3 (3%) samples showed a high level of parasitemia with *Plasmodium falciparum*. The 3 corresponding patients had fever with no apparent source. Malaria was suspected in 2 of the patients by their clinical symptoms; the third patient was thought to have typhoid and was treated with trimethoprim-sulfamethoxazole.

Two patients (2%) had acute, secondary dengue infections that were confirmed as positive by both IgM and IgG serologic tests. Both patients had a chief report of fever with no source, but malaria was suspected by the attending physician, and 1 patient was treated with chloroquine. We were not able identify dengue viral particles in the serum specimens of these patients. However, 79 patients (68%) were positive for anti-dengue IgG, which suggests a high level of flavivirus transmission in this area in the recent past (Figure).

**Figure:**
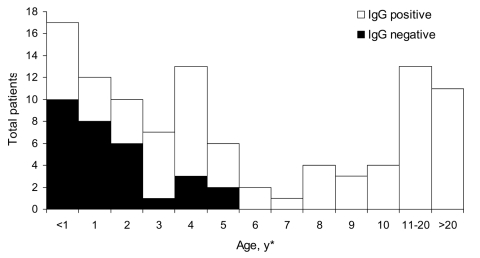
Results of immunoglobulin G (IgG) ELISA for antiflavivirus antibodies among patients exhibiting fever, Gonaïves, Haiti, October 2004 (n = 105). *Exact ages are not available for 11 patients.

Two patients (2%) had MIA results consistent with acute West Nile virus infection. The results were confirmed by PRNT (Table). Both patients were febrile in the clinic; 1 was a 13-year-old boy and the other was an infant girl <1 year of age. In addition to fever, the 13-year-old reported headache and abdominal pain, while cough was reported in the infant. Acute malaria was clinically diagnosed in both patients. The older child received chloroquine, while the younger child received only acetaminophen for fever control.

**Table:** Endpoints for 90% plaque reduction neutralization tests for patients with acute West Nile virus (WNV) infection, Gonaïves, Haiti, 2004*

Age	Sex	DEN-1	DEN-2	DEN-3	DEN-4	SLE	WNV
13 y	Male	<100	<100	<100	<100	<100	200
<1 y	Female	<100	<100	<100	<100	<100	800

*DEN, dengue; SLE, Saint Louis encephalitis virus.

## Conclusions

This surveillance program was established to assess the incidence of vectorborne diseases in the wake of Hurricane Jeanne. A total of 116 acutely febrile patients had blood drawn to determine whether a mosquitoborne disease was the etiologic agent of fever. An outbreak of mosquitoborne disease was not detected during the period of surveillance. Our data are consistent with previously published reports, which indicate that the incidence of arboviral infections rarely increases after water-related disasters (e.g., floods, hurricanes) (*7**−**9*). However, malaria outbreaks are common in such settings (*9**−**11*).

Despite the absence of an outbreak, our surveillance did identify the ongoing transmission of 3 mosquitoborne pathogens. Specifically, we diagnosed 3 cases of acute malaria, 2 cases of acute dengue, and 2 cases of acute West Nile virus infection. We also detected a high seroprevalence of dengue infections in children, which suggests substantial local dengue transmission in the Gonaïves area in the recent past.

The high seroprevalence of dengue and the low smear-positive rate of malaria from our surveillance were consistent with previously reported studies in this region of Haiti (*12*,*13*). The identification of 2 patients with positive West Nile virus results in Haiti is new. The only other human West Nile virus infections identified in the Caribbean Basin were 1 case reported in a Cayman Islands resident in 2001 (*14*) and 2 cases reported in Cuba, 1 in 2003 and the other in 2004 (*15*). This finding is not unexpected, however, because Komar et al. have identified West Nile virus in bird species native to the Dominican Republic, located to the east of Haiti on the island of Hispaniola.

The fact that the rate of West Nile virus infection was equal to the rate of acute dengue infection among our participants is of concern. Moreover, because both viruses can cause a nonlocalizing fever, the potential for confusion with malaria exists. Differentiating the cause of acute nonlocalizing febrile illnesses by examining malaria smears before initiating therapy, especially in an area with a history of low smear positivity, is therefore important.
